# In Utero Exposure of Hyperlipidemic Mice to Diesel Exhaust: Lack of Effects on Atherosclerosis in Adult Offspring Fed a Regular Chow Diet

**DOI:** 10.1007/s12012-017-9399-x

**Published:** 2017-01-17

**Authors:** Jenna Harrigan, Divya Ravi, Jerry Ricks, Michael E. Rosenfeld

**Affiliations:** 1 Program in Nutritional Sciences, University of Washington, Box 358050, Seattle, WA 98109-4714 USA; 2 Department of Environmental and Occupational Health Sciences, University of Washington, Seattle, WA USA; 3 Department of Pathology, University of Washington, Seattle, WA USA

**Keywords:** Atherosclerosis, Apo E-deficient mice, Intrauterine stress, Air pollution, Diesel exhaust

## Abstract

Uterine stress is associated with an increased risk of later life metabolic diseases. In this study, we investigated the effect of diesel exhaust (DE) exposure in utero on adult susceptibility to atherosclerosis in genetically hyperlipidemic mice. Pregnant apolipoprotein E-deficient mice received either DE exposure (~250–300 μg/m^3^ PM_2.5_ for 6 h/day, 5 days/week) or filtered air (FA) throughout gestation. Treatment effects on litter size and gender distribution were recorded. Plasma cholesterol and triglycerides were measured at 8, 12 and 16 weeks of age. Urinary 8-isoprostane and liver 8-hydroxy-deoxyguanosine levels were measured at killing at 16 weeks of age. Expression of the antioxidant genes heme oxygenase-1 and the glutamate-cysteine ligase modifier and catalytic subunits were measured in the lung, liver and aorta. The average area and frequency of atherosclerotic lesions were measured in the aortic sinus and innominate arteries. There were significantly smaller litters and higher postnatal mortality in the DE-exposed mice. There were no significant differences in plasma lipids or lipoprotein profiles, expression of antioxidant genes or markers of oxidative stress between treatment groups. There were also no significant differences in average atherosclerotic lesion area in the aortic sinus or innominate arteries of the DE and FA groups although there was a higher frequency of lesions in the DE-exposed group. Our study indicates that in utero DE exposure does not influence later life lipoprotein metabolism, redox homeostasis or the risk of developing larger atherosclerotic lesions.

## Introduction

Barker and colleagues first established the concept of the “fetal origin of disease” whereby they demonstrated that maternal malnutrition during pregnancy caused intrauterine stress and growth retardation with reduced birth weight. This was followed by a rapid catch-up in growth that was ultimately associated with obesity, hypertension and cardiovascular disease in adulthood [1]. Intrauterine stress can also be induced by other environmental factors such as exposure to air pollution, and it has been reported that maternal exposure to air pollution particulate matter (PM) is associated with reduced birth weight [2]. Exposure of pregnant mice to diesel exhaust (DE), a major contributor to air pollution particulates, causes placental and fetal inflammation and is associated with obesity in adulthood [3]. In a series of recent studies conducted at the same DE exposure facility that was utilized in the current study, Weldy et al. [4–6] reported that in utero DE exposure of C57Bl/6 mice induced uterine stress, placental inflammation, fetal resorption and increased susceptibility to aortic constriction-induced heart failure in adulthood. Exposure to air pollution, especially fine and ultrafine particulates, is a risk factor for cardiovascular disease [7]. It is thought to contribute by causing airway inflammation with spillover of pro-inflammatory cytokines, systemic oxidative stress, effects on the autonomic nervous system with altered heart rates, dysregulation of vascular tone and increased coagulation [7]. Animal studies suggest that there may also be effects of PM exposure on lipid metabolism and the development of atherosclerosis [8], the primary cause of ischemic heart disease and stroke. Hyperlipidemia during pregnancy can lead to the development of the early stages of atherosclerosis in the fetuses of both mice and humans [9, 10] and cause epigenetic alterations in utero which could influence cardiovascular risk in adults [11]. As in utero DE exposure increases the susceptibility to induced heart failure in adulthood [4] and because atherosclerosis can begin during fetal development [9], we hypothesized that in utero DE exposure of genetically hyperlipidemic mice would accelerate the spontaneous development of atherosclerosis in the offspring in early adulthood.

## Methods

This study was carried out in accordance with the Guide for the Care and Use of Laboratory Animals of the National Institutes of Health recommendations. All animal experiments were approved by the University of Washington Institutional Animal Care and Use Committee (IACUC protocol no. 2650-08).

### Experimental Design

Apolipoprotein E-deficient (apo E-/-) female and male mice of 8–16 weeks of age were bred in a modified specific pathogen-free breeding colony housed in the University of Washington South Lake Union Campus Brotman Building vivarium. At 3 different time points, sets of 7–15 mice were transferred to the Northlake DE exposure facility at the University of Washington. All adult mice were kept at the Northlake facility in Allentown caging systems (Allentown, NJ, USA) with a 12-h light/dark cycle as previously described [12]. DE was generated with a Yanmar America Corp. single-cylinder diesel engine generator set with a maximum electrical power output of 5.5 kW (Model YDG5500EV-6EI) using diesel fuel with a maximum sulfur content of 15 ppm. The highway grade diesel fuel was obtained from local fuel distributors. The load on the generator was maintained at 82% (Simplex Swift-E FT load bank). The overall DE dilution was 1:380 with heated and humidified air. The DE was aged for ~5 min in order to mimic DE aging in the atmosphere. All dilution air for the system was passed through HEPA and carbon filters. The mass concentration was monitored during exposures with a TEOM analyzer (Rupprecht & Patashnick Model 1400a) with a PM_2.5_ cyclone inlet. The PM_2.5_ concentrations measured on 4 days of sampling by the TEOM were between 250 and 300 µg/m^3^ (average 277 µg/m^3^ std dev 23 µg/m^3^). The mass median aerodynamic diameter (MMAD) was 77 nm, obtained by gravimetric analysis of samples collected with a micro-orifice uniform deposit impactor (MOUDI, MSP Model 110-NR). The average particle number concentration was 145,000 particles/cm^3^ (P-Trak Ultrafine Particle Counter, Model 8525, with specified collection of particle diameters >20 nm). The mass fraction of particle-bound polycyclic aromatic hydrocarbons (Ecochem PAS 2000) was 22 ng/µg PM_2.5_. The ratio of organic carbon to elemental carbon was 0.08, based on quartz filter samples adjusted with a concurrent dynamic blank (analyzed by Sunset Laboratories using the IMPROVE A thermo-optical reflectance method). Oxides of nitrogen concentrations were 1300 ppb NO (std dev: 370 ppb) and 50 ppb NO_2_ (std dev: 50 ppb) (analyzer: Thermo Scientific Model 42C). A more detailed description of the Northlake facility system and the composition of the DE have been published elsewhere [13].

After 1 week of adjustment to the Northlake facility, each female was placed in a cage with a male for breeding. Mice were checked each subsequent morning for visible vaginal plugs to ensure that mating took place. Upon confirmation of mating, each female was moved to a randomly assigned cage for DE or filtered air (FA) exposure. DE exposures to 250–300 µg/m^3^ PM_2.5_ were for 6 h per day for 5 days per week from the determined time of mating until birth (mouse gestation period is approximately 19–21 days). Four dams were bred a second time with two exposed to DE and two exposed to FA. Offspring remained at this facility until weaning at 4 weeks of age when they were transferred back to the Brotman Building vivarium. To ensure that the mice were exposed prenatally only, litters and their dam were moved to the FA rack on the morning following their birth. The mice were fed normal mouse chow (Lab Diet #5053) and water ad libitum. Body weights of the pups were measured weekly starting at 1 week of age until killing at 16 weeks of age.

### Plasma Collection

At 8 and 12 weeks of age, blood samples were collected from the retro-orbital sinus following a 4-h fast. Mice were anesthetized by inhalation of isoflurane, and 600–900 µl of blood was collected from each mouse. Blood was transferred immediately into a heparin tube. After approximately 1 h on ice, samples were centrifuged for 10 min at 12,000 rpm. Plasma samples were stored at −80° C.

### Killing and Tissue and Urine Sample Collection

The mice were fasted for 4 h prior to killing at 16 weeks of age. Urine samples were collected, frozen in liquid nitrogen and stored at −80 °C. Mice were anesthetized by intraperitoneal injection with 35 mg/kg of a 50/50 mix of ketamine hydrochloride and xylazine. After opening the chest cavity, blood was collected by puncture through the left ventricle with a 27-gauge needle. Plasma was obtained as above. The animals were then perfused through the left ventricle with 3–5 ml of a PBS-EDTA solution (2 mM EDTA, pH 7.4). Samples of liver, lung and aorta were extracted and snap frozen in liquid nitrogen and stored at −80° C. The heart plus ascending aorta with attached branches were immersion fixed in formalin.

### Plasma Lipid Profiles

Total cholesterol and triglycerides were measured in plasma samples from each animal collected at 8, 12 and 16 weeks of age using colorimetric kits (Sekisui Diagnostics, Wako Chemicals) according to the manufacturer’s instructions. Separate pooled plasma from the females and males from each treatment group and at each time point was used to generate the lipoprotein profiles. The lipoprotein profiles were generated following separation with an AKTA purifier FPLC system with a 10/300 column (GE Healthcare). The elution was performed with a running buffer containing 0.15 M NaCl, 0.01 M Na2HPO4, pH 7.5, 0.02% NaN3. After loading 100 μl pooled plasma, the system was run with a constant flow rate of 0.5 ml/min. The cholesterol and triglyceride content in the collected fractions were determined as above.

### RNA Isolation and Quantitative RT-PCR

Flash frozen samples of liver, lung and the combined thoracic and abdominal aortas from three randomly selected male and three female mice per exposure group were used for DNA and RNA isolation. The DNA and RNA isolation was performed using a Qiagen DNA/RNA Mini kit (Qiagen AllPrep DNA/RNA Mini Kit). The isolated DNA/RNA was transferred to the National Institute of Environmental Health Sciences supported UW Center for Ecogenetics and Environmental Health Functional Genomics Laboratory where a fluorogenic 5′ nuclease-based assay was used to quantitate the mRNA levels of antioxidant specific genes. Briefly, reverse transcription was performed according to the manufacturer’s protocol using total RNA and the Life Technologies Invitrogen SuperScript III kit (Thermo Fisher Scientific). For gene expression measurements, 500 ng of starting total RNA was used to make the cDNAs; 2 μl of cDNA was included in a PCR that also consisted of the TaqMan Gene Expression Master Mix (Thermo Fisher Scientific). The expression levels of glyceraldehyde phosphate dehydrogenase (GAPDH), heme oxygenase-1 (HO-1), glutamate-cysteine ligase catalytic subunit (GCLc) and glutamate-cysteine ligase modifier subunit (GCLm) genes were assessed using the Life Technologies TaqMan Gene Expression Assays (primer numbers GCLm: Mm01324400_m1, GCLc: Mm00802655_m1, HMOX1: Mm00516005_m1, Thermo Fisher Scientific) mix according to the manufacturer’s protocol. Amplification and detection of PCR amplicons were performed with the ABI PRISM 7900 system (Applied Biosystems Inc) with the following PCR profile: 1 cycle of 95 °C for 10 min, 40 cycles of 95 °C for 30 s and 62 °C for 1 min. The GAPDH amplification plots derived from serial dilutions of an established reference sample were used to create a linear regression formula in order to calculate expression levels, and GAPDH gene expression levels were utilized as an internal control to normalize the data.

### Analysis of 8-Isoprostane and 8-Hydroxy-2-Deoxyguanosine

Pooled urine from male and female animals of randomly selected litters (3 from each exposure group) was analyzed for 8-isoprostane levels. Urinary 8-isoprostane was measured according to the manufacturer’s protocol (8-isoprostane ELISA kit, Detroit R&D). A DNA/RNA Oxidative Damage EIA kit was used to measure the DNA oxidation by-product 8-hydroxy-2-deoxyguanosine (8-OH-dG) (Cayman Chemical) in the same liver samples used for the mRNA analysis. The competitive immunoassay involves the binding of free 8-OH-dG to an antibody coated 96-well plate. The assay and sample concentration of 8-OH-dG was carried out as per the manufacturer’s instructions.

### Histological Analysis

The formalin-fixed aortic sinus bisected from the heart of each mouse was embedded in paraffin. Five-μm-thick serial sections were prepared using a Spencer Model 820 rotary microtome (American Optical). Tissue collection started when the aortic valve leaflets were visible and collection stopped when the aortic sinus was surpassed. Every third slide was stained using a modification of the Movat’s pentachrome stain [14]. Stained aortas were imaged using a Nikon Eclipse E400 microscope coupled with a Nikon DS-1 camera (Nikon, Chiyoda, Tokyo). NIH Elements F Package was used to view and save the images for further analysis. Tissue analysis was conducted without knowledge of the tissue treatment group. The cross-sectional area of atherosclerotic lesions was determined using the *Image*-*Pro* computer-assisted morphometric analysis application (*Image-Pro Plus*, Media Cybernetics). The middle section on each slide was analyzed for lesion area (approximately 30–50 sections/mouse).The lesion areas for each separate plaque located adjacent to the valve leaflets were summed in each measured section. The mean of these sums from each animal was used for statistical analysis (ANOVA). A subsample of the innominate arteries was embedded in paraffin, sectioned and stained with the modified Movat’s pentachrome stain, and the average lesion area was measured as described above.

## Results

As noted, exposure of pregnant C57Bl/6 mice to DE at the Northlake exposure facility caused uterine stress, fetal resorption and increased susceptibility to induced heart failure in adulthood [4]. This prior study set the stage for the current study of the effects of DE exposure of pregnant apo E-/- mice on the spontaneous development of atherosclerosis in the adult off spring.

**Fig. 1:**
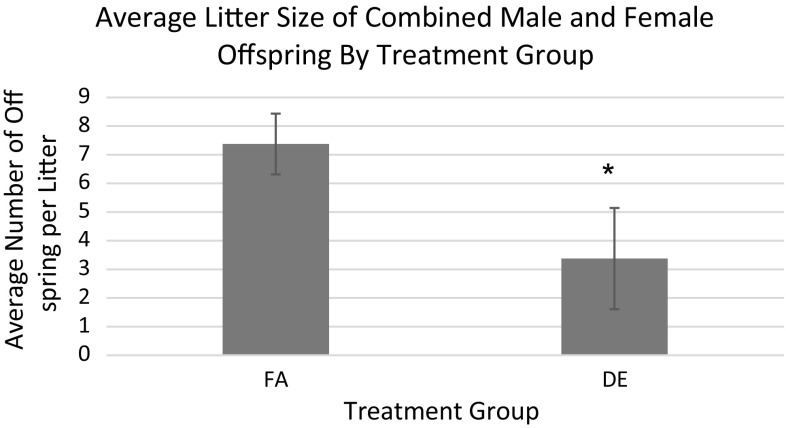
Average litter size per dam from each treatment group. Litters in which the number of offspring could not be counted due to cannibalism were not analyzed. Data shown are the mean ± standard error. *Statistically significant difference in litter sizes from the FA treatment group (*p* < 0.05). *p* = 0.0008

**Fig. 2:**
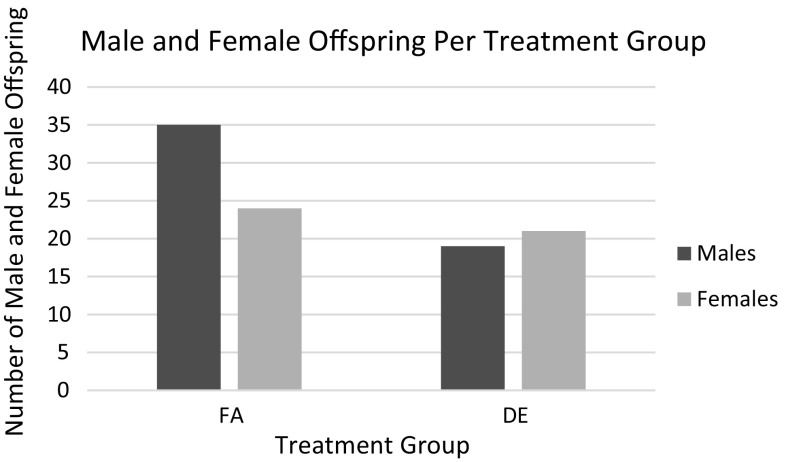
Distribution of male and female offspring born from combined litters in each treatment group. Offspring totals represent animals that survived to 4 weeks of age when sex was determined

**Fig. 3:**
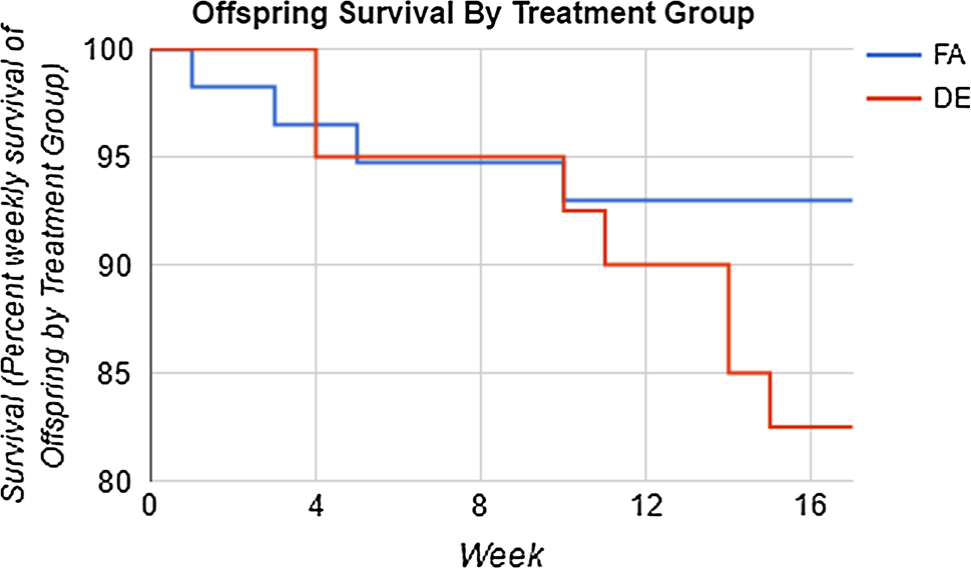
Weekly survival of combined male and female offspring among treatment groups. The mortality rate per 100 mice was: FA = 10.3, DE = 21.4

As shown in Fig. 1, there was reduced litter size in the DE-exposed apo E-/- mice. This would suggest that DE exposure of apo E-/- mice on a C57Bl/6 background caused fetal resorption as reported for non-hyperlipidemic C57Bl/6 mice by Weldy et al. [4]. The reduced litter size was associated with fewer births of male mice (Fig. 2). DE exposure during pregnancy was also associated with increased mortality in the offspring between 10 and 16 weeks of age (Fig. 3). However, there were no effects of the DE exposure during pregnancy on the rate of growth of the surviving offspring as measured by accumulated body weights (data not shown).

**Fig. 4:**
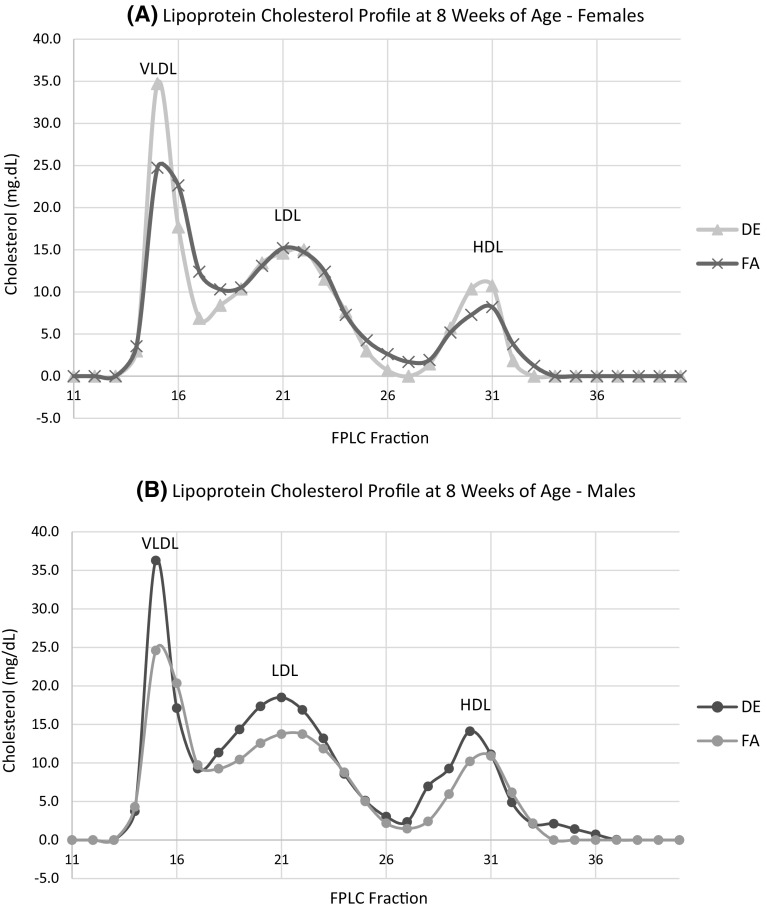
Plasma lipoprotein cholesterol content of 8-week-old apo E-/- mice. **a** Females. **b** Males. Plasma samples used for cholesterol analysis were pooled and separated into fractions using FPLC. The sum of fractions 13–17, 18–27 and 18–27 were used to represent VLDL, LDL and HDL, respectively

In utero DE exposure had no effects on either the total plasma cholesterol or triglyceride levels in the adult offspring at 8, 12 and 16 weeks of age (Table 1). There were also no effects of the DE exposure on the distribution of cholesterol or triglycerides among the different lipoprotein fractions (an example of the plasma cholesterol profiles are shown in Fig. 4).

**Table 1:** Plasma total triglycerides and cholesterol

	Filtered air	Diesel exhaust
*Total triglyceride (mg/dl)*		
8 Weeks	76.7 ± 42.7 *n* = 36	65.6 ± 25.2 *n* = 18
12 Weeks	68.5 ± 35.8 *n* = 26	65 ± 24.8 *n* = 18
16 Weeks	333.4 ± 233.6 *n* = 18	168.4 ± 124.1 *n* = 15
*Total cholesterol (mg/dl)*		
8 Weeks	253.9 ± 46.7 *n* = 35	259.5 ± 54.6 *n* = 18
12 Weeks	322.9 ± 93.5 *n* = 29	330.5 ± 134.1 *n* = 23
16 Weeks	259.4 ± 131.2 *n* = 27	367.6 ± 116.3 *n* = 16

Blood was collected via the retro-orbital sinus following a 4-h fast. Values shown are the means ± standard deviations. *n* = sample size

DE exposure of mice during pregnancy causes an increase in placental oxidative stress [4]. To determine whether there was any carryover of this placental oxidative stress to the adult offspring, we measured 8-isoprostane in the urine and 8-OH-dG in the liver of the mice at killing. There were no statistically significant effects of the in utero DE exposure on either marker of oxidative stress (Table 2). Oxidative stress also induces expression of Nrf-2-dependent antioxidant genes [15]. Thus, we also measured the levels of mRNA for the antioxidant genes HO-1, GCLc and GCLm. There were no statistically significant effects of the DE exposure on the expression of these genes in the liver, lung or aorta at 16 weeks of age (Table 3).

**Table 2:** Antioxidant gene expression

Gene/tissue	Filtered air	Diesel exhaust
*GCLc*		
Lung (*n* = 9)	1.26 ± 0.37	0.55 ± 0.05
Liver (*n* = 9)	1.15 ± 0.25	1.04 ± 0.36
Aorta (*n* = 6)	0.71 ± 0.13	0.66 ± 0.4
*GCLm*		
Lung (*n* = 10)	1.11 ± 0.22	0.78 ± 0.09
Liver (*n* = 10)	1.08 ± 0.21	1.16 ± 0.28
Aorta (*n* = 6)	0.77 ± 0.11	0.76 ± 0.43
*HMOX1*		
Lung (*n* = 10)	1.92 ± 0.96	0.24 ± 0.03
Liver (*n* = 10)	1.09 ± 0.20	0.94 ± 0.29
Aorta (*n* = 6)	0.74 ± 0.33	0.75 ± 0.46

All gene expression was normalized to GAPDH. Data shown are the mean ± SEM. Tissue samples were obtained at killing at 16 weeks of age Tissue samples from randomly selected male and female mice within each treatment group were combined. There were no significant differences (*p* < 0.05) between treatment groups

**Table 3:** Markers of oxidative stress

	DE	FA
Liver 8-hydroxyguanosine (pg/g) (*n* = 24/group)	572.9	567.3
Urine 8-isoprostane (pg/ml) (*n* = 24/group)	127.6	284.9

Urine 8-isoprostane and liver 8-hydroxyguanosine levels were measured by ELISA in randomly selected samples from each treatment group obtained at killing at 16 weeks of age. Urine samples were pooled from both females and males. There were no significant differences (*p* < 0.05) between treatment groups

**Fig. 5:**
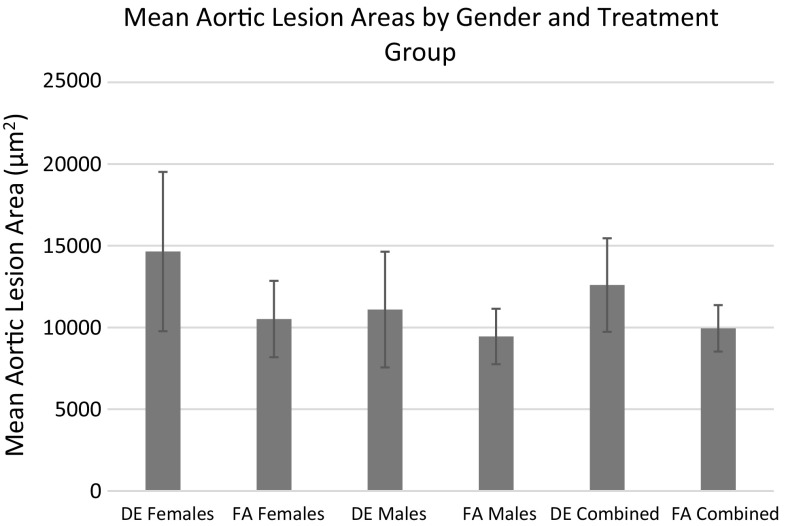
Average cross-sectional area of atherosclerotic lesions in the aortic sinus of male and female apo E-/- mice in each treatment group. Data shown are the mean ± SE (*n* = 45 FA, 21 females and 24 males, *n* = 25 DE, 11 females and 14 males). There was no significant difference (*p* < 0.05) between treatment groups

**Fig. 6:**
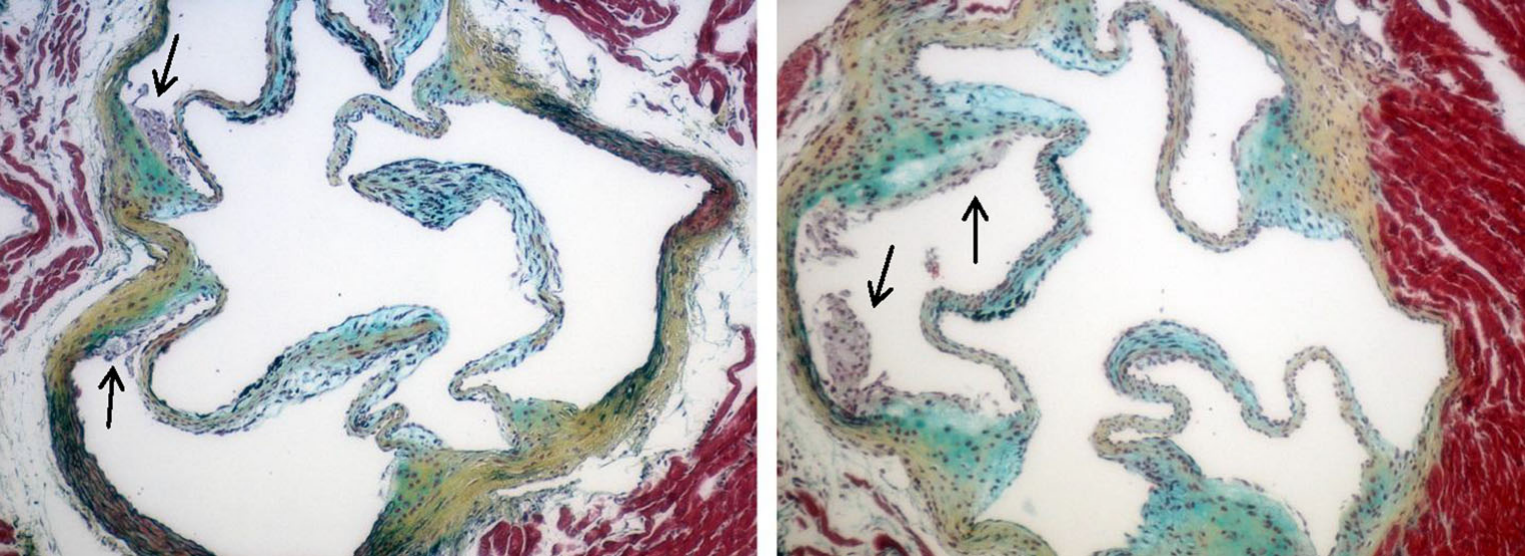
Morphology of early atherosclerotic lesions in the aortic sinus of 16-week-old chow-fed apo E-/- mice exposed to filtered air (*left*) or diesel exhaust (*right*) in utero. Five micron sections were stained with the Movat’s pentachrome stain. *Arrows* point to early fatty streaks. ×100 final magnification

There were also no effects of the in utero DE exposure on the average size or composition of early atherosclerotic lesions in either the aortic sinus (Figs. 5, 6) or the innominate arteries (Table 4). The lack of effects of the DE exposure on lesion size is in part due to the large variation in lesion size as many of the mice did not have any lesions at all at 16 weeks of age. DE exposure, however, may have affected the susceptibility to lesion formation in the innominate arteries as there were fewer mice without any lesions in the DE group (7/17) as compared to the FA group (19/27) (Table 4). A similar pattern was observed with the lesions in the aortic sinus although the effects were not as pronounced as in the innominate arteries (5/45 mice had no lesions in the FA group as compared to 2/25 mice in the DE group). A surprising observation was that male offspring of dams that had been exposed to DE multiple times (second litters) as compared to offspring from a single exposure (first litter) had smaller average lesion area in the aortic sinus (Table 5). Another surprising observation was that mice that were part of larger litters (5–9 pups) had larger average lesion area in the aortic sinus than mice that were part of smaller litters (1–4 pups) (data not shown).

**Table 4:** Effects of in utero diesel exhaust exposure on atherosclerosis in the innominate arteries of 16-week-old apo E-/- mice

No. of mice	Filtered air		Diesel exhaust	
	*N*	%	*N*	%
*Gender*				
Female	10	37	8	47
Male	17	63	9	53
*Lesion frequency*				
No. of animals with lesions	8	29	10	59
No. of animals without lesions	19	71	7	41
Average lesion area (*µ* ^2^)	3486.04 ± 2237.7		3269.02 ± 1683.4	

The frequency and mean cross-sectional area of atherosclerotic lesions in the innominate arteries were measured in a combined subset of both male and female apo E-/- mice. *N* = 5 males and 3 females for FA group. *N* = 6 males and 4 females for the DE group. Data shown for the lesion area are the mean ± the standard deviation

**Table 5:** Litter size (A) and lesion area in the aortic sinus (B) as a result of single versus multiple DE exposures

Exposure #	DE litter size	DE females in each litter/males in each litter	FA litter size	FA females in each litter/males in each litter	
1	5	3/2	6	3/3	
1	4	3/1	6	0/6	
1	4	3/1	7	3/4	
1	6	1/5	8	4/4	
1	7	3/4	6	3/3	
1	4	4/0	8	2/6	
2	4	2/2	8	5/3	
2	6	3/3	8	3/5	

First-generation DE total (M and F) (*n* = 30)	First-generation DE F (*n* = 15)	First-generation DE M (*n* = 15)	First-generation FA total (M and F) (*n* = 41)	First-generation FA F (*n* = 15)	First-generation FA M (*n* = 26)
9781.9	5708.0	13,855.8	14,311.3	19,173.6	9449.0
Second-generation DE total (M and F) (*n* = 10)	Second-generation DE F (*n* = 5)	Second-generation DE M (*n* = 5)	Second-generation FA total (M and F) (*n* = 16)	Second-generation FA F (*n* = 8)	Second-generation FA M (*n* = 8)
14,387.8	20,067.0	8708.7	5457.0	5188.3	5725.6

Lesion area = µm^2^, *DE* diesel exhaust, *FA* filtered air, *F* female, *M* male, *n* = sample size

## Discussion

DE-exposed apo E-/- dams had smaller litter sizes (Fig. 1), indicating that DE exposure may have caused embryo resorption or fetal death. This is in keeping with the report by Weldy et al. [4] of embryo resorption in C57Bl/6 dams exposed to DE in the same facility as the current study and with a previously reported epidemiologic study showing elevated intrauterine mortality associated with ambient air pollution exposure [16]. Additional evidence from human studies supports the role of systemic and placental oxidative stress in the pathology of spontaneous abortion [17]. Oxidant-induced endothelial damage and impaired placental vascularization are thought to be key players in recurrent pregnancy loss [17]. DE-exposed dams also had fewer males (Fig. 2; Table 5). Low ratios of males to females among live births and higher male fetal deaths have been observed in human cohorts after acute exposure to environmental stressors [18]. There were also more deaths in DE-exposed offspring between 10 and 16 weeks of age (Fig. 3). It is unclear as to the cause of death of these mice as necropsies were not performed because the deaths occurred many hours prior to discovery.

This is the first study that we are aware of to test the hypothesis that in utero exposure to DE in hyperlipidemic mice will accelerate the spontaneous development of atherosclerotic lesions in adulthood. Counter to this hypothesis, there were no effects of the in utero DE exposure on the average lesion area in the aortic sinus or the innominate arteries when the mice were fed a regular chow diet. Follow-up studies are needed to evaluate the effects of in utero DE exposure in fat fed apo E-/- mice. The current results likely reflect the lack of differences in plasma lipids and markers of oxidative stress. Furthermore, the observed survival differences among the treatment groups may have confounded the mean lesion area results if only the healthiest animals with smaller lesions were included in the lesion analysis. The lack of statistically significant differences in mean lesion areas may also be due to the small sample sizes and large standard deviations as many of the mice had no lesions at all at 16 weeks of age. It is conceivable that differences in atherosclerosis progression and composition would have been evident had we killed the mice at a later time point. We chose to evaluate the effects of in utero DE exposure on the very earliest stage of atherosclerosis because we thought it would be more likely to show carry over effects of the in utero DE exposure in the younger mice and in lesions that are predominantly composed of macrophage-derived foam cells. Another factor that may have contributed to the lack of differences was the relatively short exposure period ending at birth instead of at weaning. Previous in utero DE studies reported greater cardiovascular effects after a longer maternal exposure that included lactation [5]. However, in utero exposure to arsenic only during pregnancy has been reported to accelerate the development of atherosclerosis in apo E-/- mice [19]. It is also conceivable that the exposure of the pregnant dams only during the normal work week (5-day exposure followed by weekends off) could have missed critical windows of development of the cardiovascular system and may have also contributed to the lack of differences between the treatment groups.

The increased frequency of lesions in the innominate arteries of the in utero DE-exposed offspring suggests that there may have been an increased susceptibility to lesion initiation at this site but that once the disease process had started, the rate of macrophage lipid accumulation was comparable given that the plasma lipids were equivalent. It is unclear why there was not a comparable difference in lesion frequency in the aortic sinus lesions. Previous studies in apo E-/- and low-density lipoprotein receptor-deficient mice exposed to concentrated ambient particles reported increases in plasma TC and TG and decreases in HDL cholesterol [20, 21]. Furthermore, maternal sterol metabolism contributes to fetal cholesterol levels in a number of experimental animal models [22]. Thus, it was conceivable that the DE group could have had a more pro-atherogenic lipid profile at each time point. However, there were no differences in plasma lipid levels or lipoprotein profiles in the DE and FA groups (Table 1; Fig. 4) indicating that any elevating effect on plasma lipids of the DE exposure in utero had normalized by the time plasma lipids were first measured at 8 weeks of age. The lack of effects on plasma lipids is also in agreement with other exposure studies in apo E-/- mice on a chow diet, including a previous study conducted with mice from the same breeding colony and exposure facility [23, 24]. We had also previously shown that a short-term exposure of apo E-/- mice to DE caused a loss of the anti-inflammatory and antioxidant properties of HDL that are thought to be athero-protective [12]. However, any negative effects of the DE exposure on the properties of the maternal HDL apparently did not transfer to the offspring. Furthermore, it is unclear whether intact maternal HDL is transported across the placenta despite the fact that trophoblasts and placental endothelial cells express the HDL receptor SR-B1 [25]. It is possible that feeding a cholesterol-rich diet in place of the standard chow diet to the dams during pregnancy and to the offspring following weaning could have magnified an elevating effect of in utero DE exposure on postnatal plasma lipids. However, we chose to feed the mice a standard mouse chow because we were concerned that very high plasma lipid levels as induced by a high cholesterol diet would mask any modest independent effects of the in utero DE exposure on the development of atherosclerosis in adulthood. Follow-up studies are needed to evaluate the effects of in utero DE exposure in fat fed apo E-/- mice. It would also be interesting to evaluate whether in utero DE exposure leads to aggravated responses to other irritants in adulthood such as second hand smoke or house dust mite-induced asthma.

There was a trend toward reduced gene expression (HO-1, GCLm, GCLc) and oxidative stress markers (8-isoprostane and 8-OH-dG) in the DE-exposed mice at 16 weeks that might indicate a compensatory response to oxidative stress at an earlier time point. However, the lack of any statistically significant carry over effect of the DE-induced oxidative stress in the placenta to the expression of the antioxidant genes or formation of markers of oxidative stress at 16 weeks of age is not surprising and suggests that any epigenetic effects on gene expression in the offspring were not prolonged and did not contribute to the development of atherosclerosis. This is in contrast to the reported carry over effect of the in utero DE exposure on the susceptibility to induced heart failure [4, 5]. It is possible that there are more prolonged effects of the DE exposure on fetal cardiac myocytes than on fetal vascular cells.

In conclusion, although DE exposure during pregnancy can cause intrauterine stress and placental inflammation in mice, it does not influence later life lipoprotein metabolism and redox homeostasis and does not contribute to the development of spontaneous atherosclerosis in adulthood. Follow-up studies are indicated to verify that in utero DE exposure may increase the susceptibility to lesion initiation at predilection sites such as the innominate arteries.
